# Telomere Length Change in a Multidomain Lifestyle Intervention to Prevent Cognitive Decline: A Randomized Clinical Trial

**DOI:** 10.1093/gerona/glaa279

**Published:** 2020-11-11

**Authors:** Shireen Sindi, Alina Solomon, Ingemar Kåreholt, Iiris Hovatta, Riitta Antikainen, Tuomo Hänninen, Esko Levälahti, Tiina Laatikainen, Jenni Lehtisalo, Jaana Lindström, Teemu Paajanen, Markku Peltonen, Dharma Singh Khalsa, Benjamin Wolozin, Timo Strandberg, Jaakko Tuomilehto, Hilkka Soininen, Tiia Ngandu, Miia Kivipelto, FINGER Study Group

**Affiliations:** 1 Division of Clinical Geriatrics, Centre for Alzheimer Research, Karolinska Institutet, Stockholm, Sweden; 2 Ageing Epidemiology (AGE) Research Unit, School of Public Health, Imperial College London, London, UK; 3 Institute of Clinical Medicine, Neurology, University of Eastern Finland, Kuopio, Finland; 4 Theme Aging, Karolinska University Hospital, Stockholm, Sweden; 5 Aging Research Center, Karolinska Institutet and Stockholm University, Stockholm, Sweden; 6 Institute of Gerontology, School of Health and Welfare, Aging Research Network—Jönköping (ARN-J), Jönköping University, Jönköping, Sweden; 7 SleepWell Research Program, Faculty of Medicine, University of Helsinki, Helsinki, Finland; 8 Department of Psychology and Logopedics, Medicum, University of Helsinki, Helsinki, Finland; 9 Center for Life Course Health Research, University of Oulu, Oulu, Finland; 10 Medical Research Center Oulu, Oulu University Hospital, Oulu, Finland; 11 Neurocenter, Neurology, Kuopio University Hospital, Kuopio, Finland; 12 Public Health and Welfare Department, Finnish Institute for Health and Welfare, Helsinki, Finland; 13 Institute of Public Health and Clinical Nutrition, University of Eastern Finland, Kuopio, Finland; 14 Finnish Institute of Occupational Health, Helsinki, Finland; 15 Alzheimer’s Research and Prevention Foundation, Tucson, AZ, USA; 16 Department of Pharmacology and Neurology, Boston University School of Medicine, Boston, MA, USA; 17 University of Helsinki, Clinicum, and Helsinki University Hospital, Helsinki, Finland; 18 Department of Public Health, University of Helsinki, Helsinki, Finland; 19 South Ostrobothnia Central Hospital, Seinäjoki, Finland

**Keywords:** Behavioral intervention, Dementia prevention, Older adults, Telomeres

## Abstract

**Background:**

Shorter leukocyte telomere length (LTL) is associated with aging and dementia. Impact of lifestyle changes on LTL, and relation to cognition and genetic susceptibility for dementia, has not been investigated in randomized controlled trials (RCTs).

**Methods:**

Finnish Geriatric Intervention Study to Prevent Cognitive Impairment and Disability is a 2-year RCT enrolling 1260 participants at risk for dementia from the general population, aged 60–77 years, randomly assigned (1:1) to multidomain lifestyle intervention or control group. The primary outcome was cognitive change (Neuropsychological Test Battery *z*-score). Relative LTL was measured using quantitative real-time polymerase chain reaction (trial registration: NCT01041989).

**Results:**

This exploratory LTL substudy included 756 participants (377 intervention, 379 control) with baseline and 24-month LTL measurements. The mean annual LTL change (*SD*) was −0.016 (0.19) in the intervention group and −0.023 (0.17) in the control group. Between-group difference was nonsignificant (unstandardized β-coefficient 0.007, 95% CI −0.015 to 0.030). Interaction analyses indicated better LTL maintenance among apolipoprotein E (APOE)-ε4 carriers versus noncarriers: 0.054 (95% CI 0.007 to 0.102); younger versus older participants: −0.005 (95% CI −0.010 to −0.001); and those with more versus less healthy lifestyle changes: 0.047 (95% CI 0.005 to 0.089). Cognitive intervention benefits were more pronounced among participants with better LTL maintenance for executive functioning (0.227, 95% CI 0.057 to 0.396) and long-term memory (0.257, 95% CI 0.024 to 0.489), with a similar trend for Neuropsychological Test Battery total score (0.127, 95% CI −0.011 to 0.264).

**Conclusions:**

This is the first large RCT showing that a multidomain lifestyle intervention facilitated LTL maintenance among subgroups of older people at risk for dementia, including APOE-ε4 carriers. LTL maintenance was associated with more pronounced cognitive intervention benefits.

**Clinical Trials Registration Number:**

NCT01041989

Telomeres are segments of DNA located at the ends of eukaryotic chromosomes, with an essential role in protecting chromosomes from damage and ensuring genome stability (1). Telomere shortening during cell divisions is counteracted by a complex maintenance system including the enzyme telomerase (1). Shorter telomere length is a biomarker of cellular aging and may be involved in the pathophysiology of aging-related conditions including dementia and cognitive impairment (2–8). Although telomere length and its age-dependent attrition rate are heritable, environmental factors may contribute to ≈72% of the variability in telomere length (9). Ample evidence from observational studies emphasizes the importance of a healthy lifestyle for telomere maintenance. Lifestyle factors associated with shorter telomeres include, for example, smoking, obesity, physical inactivity, and unhealthy diet (10,11).

The impact of lifestyle interventions on telomere length has so far been investigated in a small number of trials focusing primarily on other conditions and predominantly targeting unimodal interventions. A 6-month randomized controlled pilot trial in 33 older individuals with mild cognitive impairment suggested that telomere shortening was attenuated by ω-3 polyunsaturated fatty acid supplementation (12). In the randomized controlled Finnish Diabetes Prevention Study, a multidomain lifestyle intervention including weight loss, diet, and exercise did not have a significant effect on 5-year leukocyte telomere length (LTL) change compared with controls in 311 middle-aged participants with impaired glucose tolerance; LTL increased in both intervention and control groups (13). Some randomized controlled trials of meditation or mindfulness for stress reduction indicated improved telomerase activity (14). Comprehensive lifestyle intervention including diet, exercise, stress management, and increased social support was reported to increase LTL 5 years after the intervention in 10 men with low-risk prostate cancer compared with 25 controls (15). However, the majority of multidomain lifestyle interventions aiming to prevent or delay cognitive impairment have not included LTL as an outcome (16). To date, no trials have assessed whether multidomain lifestyle interventions affect LTL among older individuals at risk for dementia.

The Finnish Geriatric Intervention Study to Prevent Cognitive Impairment and Disability (FINGER) has previously reported cognitive benefits for a 2-year multidomain lifestyle intervention versus health advice control in 1260 older individuals at risk for dementia (17). The FINGER exploratory LTL substudy aimed to investigate (a) impact of the lifestyle intervention on change in LTL; (b) potential effect modification by the apolipoprotein E (APOE)-ε4 allele, age, sex, and lifestyle changes; and (c) associations between intervention benefits on cognition and change in LTL. The hypothesis was that the intervention would attenuate LTL shortening, and that better LTL maintenance would be associated with cognitive benefits.

## Method

### Study Design

FINGER is a 2-year multidomain randomized controlled trial carried out in 6 sites in Finland and enrolled at-risk participants from the general population. The trial protocol and primary findings have been previously described (17,18). FINGER was approved by the coordinating ethics committee of the Hospital District of Helsinki and Uusimaa. Participants gave written informed consent at the screening and baseline visits.

### Participants

**Figure 1.**
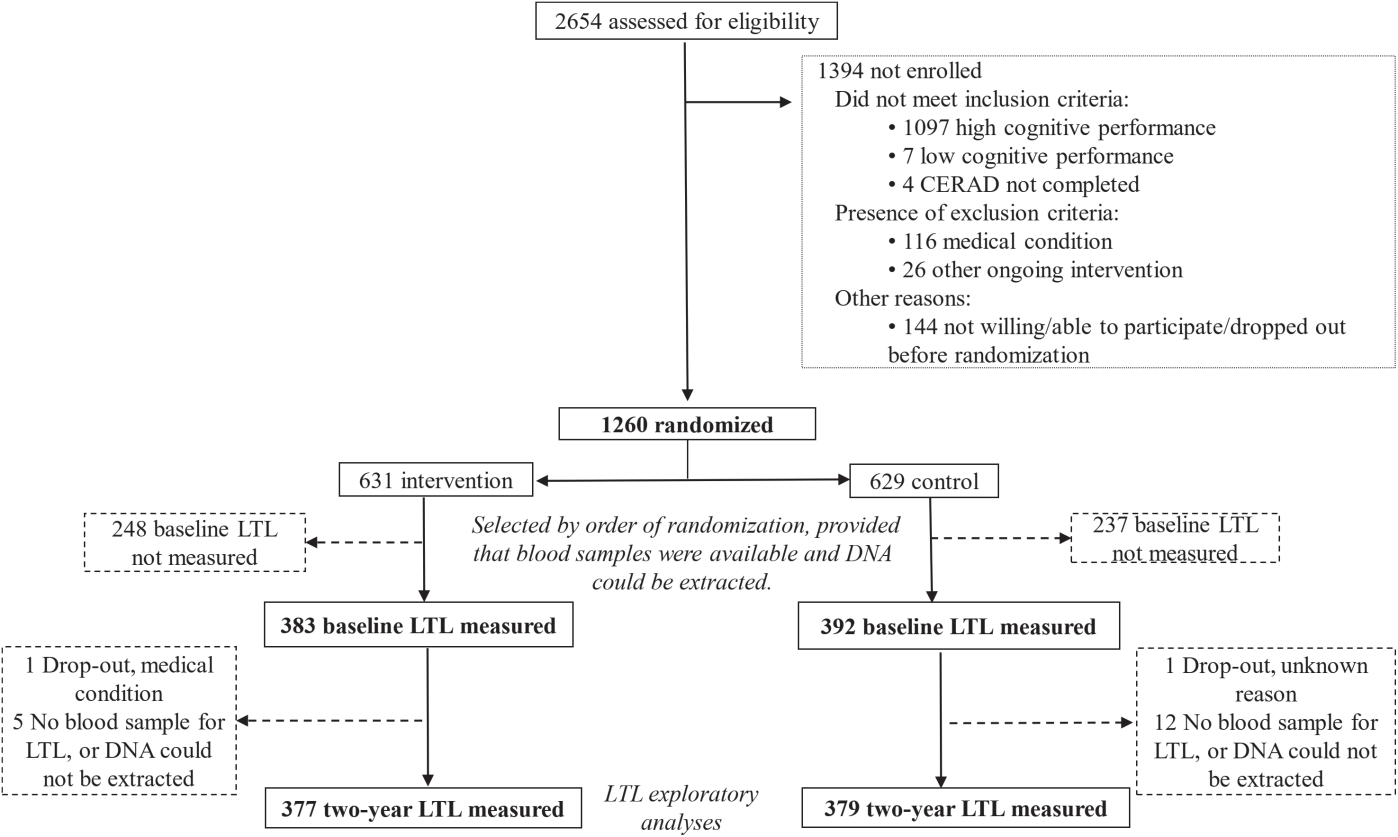
Trial profile for the exploratory LTL substudy. CERAD = Consortium to Establish a Registry for Alzheimer’s disease; LTL = relative leucocyte telomere length.

This exploratory LTL substudy included 756 of the 1260 trial participants with LTL measurements at both baseline and 2-year visit (377 in the intervention group and 379 in the control group, Figure 1). The LTL subpopulation was selected according to the order of randomization (the first 800 participants randomized), provided that blood samples were available, and DNA could be extracted. FINGER participants were recruited from former nonintervention population-based surveys. Eligibility criteria included age 60–77 years and Cardiovascular Risk Factors, Aging and Dementia (CAIDE) risk score ≥6 points (19). Cognitive screening using the Consortium to Establish a Registry for Alzheimer’s Disease battery (20) selected individuals with cognitive performance at the mean level or slightly lower than expected for age according to Finnish population norms (17). Exclusion criteria were previously diagnosed dementia, suspected dementia following clinical assessment at the screening visit, Mini-Mental State Examination less than 20 points, disorders affecting safe participation/cooperation, severe loss of sensory capacities, and concurrent participation in another intervention trial.

### Randomization and Masking

FINGER participants were randomly assigned to the multidomain intervention or the regular health advice (control) group in a 1:1 ratio. Allocations were computer-generated in blocks of 4 (2 individuals randomly allocated to each group) at each site after the baseline assessment by the study nurse. Outcome assessors and lab technicians analyzing biological samples were blinded to allocation and were not involved in intervention tasks.

### Procedures

The intervention protocol has been described in detail (17,18). In brief, the control group received regular health advice according to established guidelines. The multidomain lifestyle intervention included 4 components. The *nutritional component* based on the Finnish Nutrition Recommendations was carried out by study nutritionists in individual and group sessions with the participants. The *exercise training program* followed international guidelines, was led by study physiotherapists, and included aerobic training as well as strength and balance training at the gym (17). *Cognitive training* included psychologist-led group sessions and individual computer-based training (web-based in-house developed computer program adapted from previous trial protocols (21)). *Management of metabolic and vascular risk factors* followed national evidence-based guidelines and included additional meetings with the study nurse and physician (body mass index, blood pressure, hip and waist circumference measurements, physical examinations, and lifestyle recommendations (17)).

### Outcomes

#### LTL measurement

Relative LTL (exploratory outcome) was measured from DNA extracted from peripheral blood collected at baseline and 2-year visits. As a relative measure, LTL does not have a specific measurement unit.

Blood samples for DNA were collected in ethylenediaminetetraacetic acid (EDTA) tubes, tubes were turned a few times and then immediately stored at −20°C at the study centers. The blood samples were shipped monthly in dry ice to the laboratory at the Finnish Institute for Health and Welfare, where they were stored at −20°C until the time of DNA extraction. Genomic DNA was extracted from venous blood samples with Chemagic MSM1 from PerkinElmer using magnetic beads according to the manufacturer’s protocol. Prior to genotyping, DNA quality was determined using NanoDrop and PicoGreen, and DNA concentration normalized to 50 ng/µL.

Relative LTL measurements and quality control were conducted at the laboratory of Professor Iiris Hovatta at the Molecular and Integrative Biosciences Research Program, University of Helsinki, Finland.

Samples from each participant (baseline and follow-up) were assayed on the same 384-well plate. There was a random distribution between plates, but ensuring that the age, sex, and group allocation of participants (intervention vs control) were similar across plates. A quantitative polymerase chain reaction (qPCR)-based method was used as described previously (22,23), with β-hemoglobin (Hgb) as a single copy reference gene. Separate reactions for telomere and Hgb reaction were carried out in paired 384-well plates in which matched sample well positions were used. Ten nanograms of DNA were used for each reaction, performed in triplicate. Every plate included a 7-point standard curve, which was used to create a standard curve and to perform absolute quantification of each sample. Samples and standard dilutions were transferred into the plates using a multichannel pipet and dried overnight at room temperature. Specific reaction mix for telomere reaction included 270 nM tel1b primer (5′-CGGTTT(GTTTGG)5GTT-3′) and 900 nM tel2b primer (5′-GGCTTG(CCTTAC)5CCT-3′), 0.2X SYBR Green I (Invitrogen), 5 mM DTT (Sigma–Aldrich), 1% DMSO (Sigma–Aldrich), 0.2 mM of each dNTP (Fermentas), and 1.25 U AmpliTaq Gold DNA polymerase (Applied Biosystems) in a total volume of 15 µL AmpliTaq Gold Buffer II supplemented with 1.5 mM MgCl2. Hgb reaction mix included 300 nM Hgb1 primer (5′-GCTTCTGACACAACTGTGTTCACTAGC-3′) and Hgb2 primer (5′-CACCAACTTCATCCACGTTCACC-3′) in a total volume of 15 µL of iQ SyBrGreen supermix (Bio-Rad). Cycling conditions for telomere amplification were 10 minutes at 95°C followed by 25 cycles at 95°C for 15 seconds and 54°C for 2 minutes with signal acquisition. Cycling conditions for Hgb amplification were 95°C for 10 minutes followed by 35 cycles at 95°C for 15 seconds, 58°C for 20 seconds, 72°C for 20 seconds with signal acquisition. Reactions were performed with CFX384 Real-Time PCR Detection System (Bio-Rad). Melt-curve analysis was carried out at the end of the run to ensure specific primer binding.

Bio-Rad CFX Manager software was used to perform quality control, and samples with *SD* greater than 0.5 between triplicates were omitted from the analysis. Plate effect was controlled for by analyzing 5 genomic DNA control samples on every plate. We normalized the telomere and Hgb signal values separately to the mean of these control samples before taking the relative telomere to single-copy gene (T/S) ratio. The control samples were used for calculating the coefficient of variation value that was 8.35%.

#### Cognitive outcomes

Participants were assessed with an extended version of the Neuropsychological Test Battery (NTB) (24) at baseline, 12- and 24-month visits. The primary FINGER outcome was a change in NTB total score, a composite score based on 14 tests (calculated as *z*-scores standardized to the baseline mean and *SD*, with higher scores suggesting better performance) (18). Secondary cognitive outcomes included NTB domain *z*-scores for memory, processing speed, and executive functioning calculated as described previously (16,17).

### Statistical Analyses

The FINGER LTL exploratory substudy was planned with no available effect size references from clinical trials testing the impact of multidomain lifestyle interventions on LTL in older adults at risk for dementia. No power calculations were thus conducted specifically for the LTL substudy.

Chi-square and *t* tests were used to compare baseline characteristics of FINGER participants with and without available LTL measures, and intervention and control groups in the LTL subpopulation.

#### Intervention effects on change in relative LTL

Change in relative LTL was calculated as the difference between 2-year and baseline LTL, divided by time. Positive values indicated an observed increase, and negative values indicated a decrease in relative LTL. A linear regression model was used, with change in relative LTL as a dependent variable, and randomization group, baseline relative LTL, age, sex, study site, and healthy lifestyle change index as independent variables. Healthy lifestyle change was calculated for all participants as a composite index based on measures of diet (Recommended Finnish Diet Score (25)), exercise frequency (days per week), and cardiovascular factors (inverse of age- and sex-specific relative cardiovascular risk based on the FINRISK score (26)). *Z*-scores for each index component were standardized to the baseline mean and *SD*, and the difference between 24-month and baseline visit was calculated. The overall index was calculated as the mean *z*-score change (with higher values indicating healthier change) if data were available in at least 2 of the 3 lifestyle domains.

Potential effect modification by 4 factors highly relevant for LTL and/or cognition (APOE-ε4 allele carrier vs noncarrier status, sex, baseline age, and healthy lifestyle change index) was also investigated. For each factor, the linear regression model described above additionally included (a) APOE and randomization group × APOE interaction, (b) group × sex interaction, (c) group × age (continuous) interaction, and (d) lifestyle index (continuous) and group × index interaction.

Results are reported as unstandardized β-coefficients (95% CIs) and *p* values.

#### Change in relative LTL and change in cognition

Zero-skewness log-transformations were applied to skewed NTB components. Mixed effects regression models with maximum likelihood estimation were performed to assess the change in cognitive scores as a function of randomization group, time, LTL change, their 2-way interactions, and a group × time × LTL change interaction. All models were adjusted for age, sex, study site, baseline LTL, and healthy lifestyle change index. Results are reported as estimates from the *xtmixed* command in Stata, with 95% CI and *p* values. Group × time × LTL change interactions with *p* < .10 are also presented as graphs showing intervention effects on cognition (intervention–control difference) for different levels of change in LTL.

We use the term “intervention benefit” to refer to differences between the intervention and control groups that favored the intervention group.

Level of significance was less than 5% in all analyses. Stata software version 13 (Stata Statistical Software: Release 13; StataCorp LP, College Station, TX) was used.

The FINGER trial is registered with ClinicalTrials.gov, number NCT01041989.

## Results

Between September 7, 2009 and November 24, 2011, 2654 individuals were screened and 1260 were randomly assigned to the intensive intervention group (*n* = 631) or control group (*n* = 629). The full trial profile has been previously described (17). There were no significant differences in baseline characteristics between intervention and control groups in the LTL subpopulation (Table 1). The LTL subpopulation (*n* = 756) had a higher education level (*p* = .039), lower systolic blood pressure (*p* = .003), and better cognitive performance on the total NTB (*p* = .001), executive functioning (*p* ≤ .001), and processing speed (*p* = .035) domains compared with the rest of the FINGER participants (*n* = 504; Supplementary Table S1).

**Table 1.** Baseline Characteristics of Participants in the FINGER LTL Exploratory Substudy

	Total	Intervention	Control
Characteristics at Baseline	*n*	*n* = 377	*n* = 379
Demographic characteristics			
Age at the baseline visit (years)	756	69.4 ± 4.6	69.0 ± 4.8
Sex (women, %)	756	164 (43.5)	188 (49.6)
Education (years)	755	10.1 ± (3.4)	10.2 ± (3.4)
Baseline leukocyte telomere length (LTL)	756	1.06 ± 0.3	1.06 ± 0.3
APOE-ε4 carriers	724	111(31.1)	133(36.2)
Vascular factors			
Systolic blood pressure (mmHg)	747	139.2 ± 16.7	138.7 ± 15.8
Diastolic blood pressure (mmHg)	747	80.1 ± 9.8	80.2 ± 9.2
Serum total cholesterol (mmol/L)	754	5.1 ± 1.0	5.2 ± 1.0
Serum HDL-cholesterol (mmol/L)	754	1.4 ± 0.4	1.5 ± 0.4
Fasting plasma glucose (mmol/L)	756	6.1 ± 0.8	6.1 ± 0.9
Body mass index (kg/m^2^)	747	28.4 ± 4.7	27.9 ± 4.8
History of hypertension (%)	747	250 (67.0)	231 (61.8)
History of diabetes (%)	753	54 (14.4)	47 (12.4)
Lifestyle factors			
Physical activity 2 or more times/week (%)	750	272 (72.5)	268 (71.5)
Current smokers (%)	754	40 (10.6)	27 (7.2)
Alcohol drinking at least once/week (%)	751	179 (47.1)	164 (43.7)
Fish intake at least twice/week (%)	752	204 (54.3)	188 (50.0)
Daily intake of vegetables (%)	754	239 (63.6)	241 (63.8)
Recommended Finnish Diet Score points	752	12.8 ± 3.4	12.7 ± 3.3
Baseline cognition*			
NTB total score	755	0.00 ± 0.6	0.07 ± 0.6
Executive functioning	755	−0.00 ± 0.8	0.08 ± 0.8
Processing speed	755	−0.00 ± 0.8	0.08 ± 0.8
Memory	755	−0.01 ± 0.7	0.07 ± 0.6
Long-term memory	743	−0.02 ± 0.8	0.07 ± 0.7

*Notes:* FINGER = Finnish Geriatric Intervention Study to Prevent Cognitive Impairment and Disability; NTB = Neuropsychological Test Battery. Values are means ± *SD* unless otherwise specified. Differences between intervention and control groups were analyzed with chi-square and *t* tests as appropriate.

*Scores on the NTB total score, executive functioning, processing speed, memory, and long-term memory are mean values of *z*-scores of the cognitive tests included in each cognitive outcome. Higher scores indicate better performance.

Mean relative LTL (*SD*) at baseline was 1.075 (0.325) for participants aged 60–70 years and 1.042 (0.338) for participants aged 70–77 years. Because there is no “general reference scale” for the size of change in relative LTL values over time, and LTL decreases with age, these mean baseline values per age decade are provided as reference.

### FINGER Intervention Effects on Change in Relative LTL

On average, relative LTL decreased over time. Observed mean annual LTL change (*SD*) was −0.016 (0.19) in the intervention and −0.023 (0.17) in the control group (unadjusted *p* = .58). Increase in relative LTL was observed in 45.4% of the intervention group and 42.5% of the control group. No significant difference between intervention and control groups in change in relative LTL was found after adjusting for age, sex, baseline LTL, study site, and lifestyle change index (unstandardized β-coefficient 0.007, 95% CI −0.015 to 0.030, *p* = .53). However, effect modifications on LTL maintenance by APOE-ε4 allele, age, and lifestyle changes were observed (Table 2). The intervention had significantly more pronounced effects on LTL maintenance among APOE-ε4 carriers compared with noncarriers (unstandardized β-coefficient for randomization group × APOE interaction 0.054, 95% CI 0.007–0.102, *p* = .026), younger versus older participants (unstandardized β-coefficient for group × age interaction −0.005, 95% CI −0.010 to −0.001, *p* = .031), and participants with more pronounced versus less pronounced healthy lifestyle changes (unstandardized β-coefficient for group × lifestyle interaction 0.047, 95% CI 0.005–0.089, *p* = .029; Table 2). No significant effect modification by sex was found (group × sex interaction −0.019, 95% CI −0.065 to 0.026, *p* = .40). Findings were similar when analyses were re-run without adjustment for baseline LTL (Supplementary Table S2).

**Table 2.** FINGER Intervention Effect on Change in LTL—Impact of APOE-ε4 Allele, Age, Sex, and Healthy Lifestyle Changes

	Difference Between Intervention and Control Groups*		Randomization Group × Factor Interaction†
Factor	Unstandardized β-coefficient (95% CI), *p* value		
APOE-ε4 allele	Carriers	**0.049 (0.006 to 0.092), *p* = .025**	**0.054 (0.007 to 0.102), *p* = .026**
	Noncarriers	−0.006 (−0.033 to 0.021), *p* = .654	
Baseline age	≤69 years	**0.035 (0.001 to 0.069), *p* = .046**	−**0.005 (**−**0.010 to** −**0.001), *p* = .031**
	>69 years	−0.017 (−0.048 to 0.013), *p* = .258	
Healthy lifestyle change index	>Mean	0.022 (−0.010 to 0.055), *p* = .179	**0.047 (0.005 to 0.089), *p* = .029**
	≤Mean	−0.007 (−0.038 to 0.023), *p* = .643	
Sex	Women	−0.004 (−0.038 to 0.031), *p* = .834	−0.019 (−0.065 to 0.026), *p* = .40
	Men	0.016 (−0.015 to 0.046), *p* = .321	

*Notes:* FINGER = Finnish Geriatric Intervention Study to Prevent Cognitive Impairment and Disability; LTL = leukocyte telomere length. Unstandardized β-coefficients (95% CIs) and *p* values are shown from linear regression models with change in relative LTL as a dependent variable, and randomization group, baseline relative LTL, age, sex, study site, and healthy lifestyle change index as independent variables. Bold font indicates *p* values less than .05.

*Positive coefficients indicate intervention benefit (intervention–control differences in LTL change favoring the intervention group). For the descriptive purpose, analyses were stratified by factor group: APOE-ε4 carriers and noncarriers; baseline age below and above the rounded mean value; healthy lifestyle change index above and below the mean value (ie, more vs less improvement); and women and men.

†Unstandardized coefficients, 95% CIs, and *p* values are shown for randomization group × factor interactions where age and healthy lifestyle change index were entered as continuous variables.

### Change in Relative LTL and Change in Cognition

**Figure 2.**
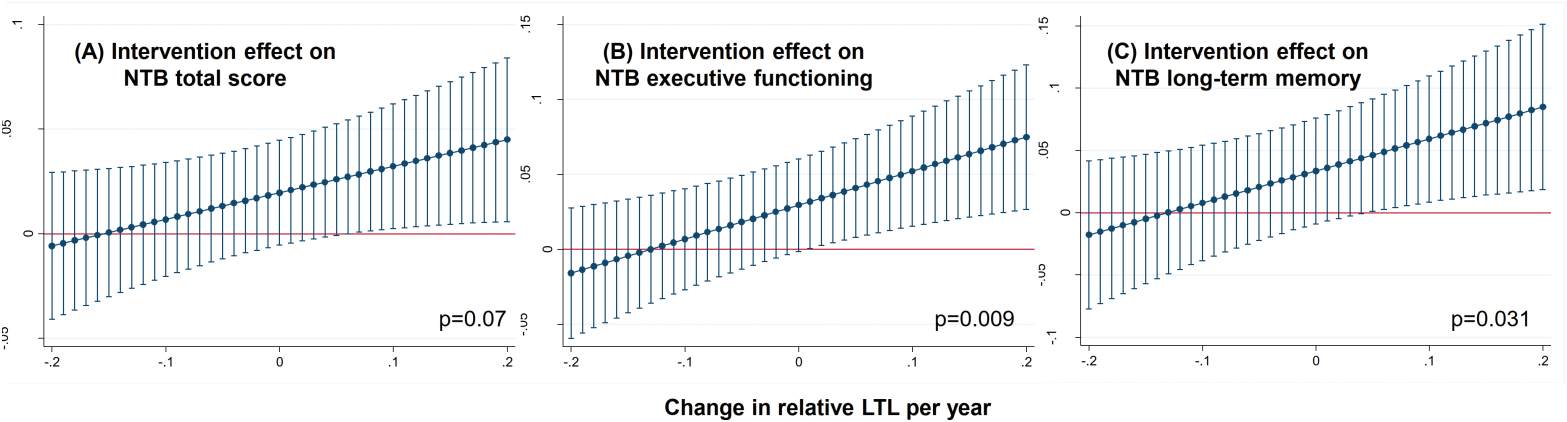
Intervention effects on cognition for different levels of change in relative leucocyte telomere length (LTL). The x-axis shows the annual change in relative LTL. The y-axis shows the FINGER intervention effect (ie, intervention–control difference) on the annual change in cognitive outcomes (standardized *z*-scores). Positive values indicate cognitive benefit (ie, the intervention effect on cognition favors the intervention group). Error bars are 95% CIs. Average marginal effects are estimated from linear mixed models including randomization group, time, change in LTL, their 2-way interactions, the group × time × change in LTL interaction, age, sex, study site, baseline LTL, and healthy lifestyle change index. An increase in cognitive benefit is seen with an increase in relative LTL (*p* values are shown for the group × time × change in LTL interaction). (A) Intervention effect on NTB score. (B) Intervention effect on NTB executive functioning. (C) Intervention effect on NTB long-term memory.

The intervention benefit on the primary cognitive outcome (NTB total score) tended to be more pronounced with increasing relative LTL (Figure 2A). The randomization group × time × LTL change interaction (95% CI) was 0.127 (−0.011 to 0.264), *p* = .070. Intervention benefits on executive functioning and long-term memory were significantly more pronounced with increasing relative LTL (Figure 2B and C). The randomization group × time × LTL change interaction (95% CI) was 0.227 (0.057–0.396), *p* = .009 for executive functioning and 0.257 (0.024–0.489), *p* = .031 for long-term memory. No significant randomization group × time × LTL change interactions were found for processing speed: −0.087 (−0.268 to 0.094), *p* = .347 or memory: 0.152 (−0.074 to 0.378), *p* = .187. Findings were similar when analyses were re-run without adjustment for baseline LTL (Supplementary Table S3).

No serious intervention-related adverse events were reported (17).

## Discussion

FINGER is so far the largest clinical trial investigating the effects of a multidomain lifestyle intervention on change in LTL and the first to relate the change in LTL to change in cognition in older adults at risk for dementia from the general population. Overall, LTL change during 2 years was not significantly different between the intervention and control groups. However, there were significant intervention benefits on LTL maintenance in APOE-ε4 carriers, younger individuals, and participants with healthier lifestyle changes during the 2-year trial. No differences were found between men and women. Intervention benefits on the primary cognitive outcome (NTB total score) were more pronounced with better LTL maintenance. Intervention benefits on long-term memory and executive functioning, but not memory or processing speed, were significantly more pronounced with better LTL maintenance.

These findings add to the sparse literature on multidomain lifestyle interventions and LTL. For ethical reasons, the FINGER control group also received advice on lifestyle, metabolic, and vascular risk factors, and this may have diluted the effects compared with a “do-nothing” control group. Mean LTL decreased during the 2-year trial, which was expected given the participants’ age. Intervention effects on LTL maintenance were also less pronounced with older age at baseline. This suggests that LTL maintenance through healthy lifestyle changes may decrease with age and thus emphasizes the importance of early interventions.

Improvement in lifestyle among FINGER participants was directly associated with LTL maintenance. This finding is consistent with previous observational studies on lifestyle and LTL, and most importantly with the notion that healthy behaviors act in synergy to affect LTL positively (27). In the Nurses’ Health Study, the combination of 5 healthy lifestyle factors (non-current smoking, maintaining optimal body weight, healthy diet, exercise, and moderate alcohol consumption) was cross-sectionally associated with longer LTL. However, associations with LTL were much weaker when each lifestyle factor was assessed separately (28). Similarly, healthy behaviors regarding diet, physical activity, and sleep have a joint effect in buffering against the deleterious impact of stress on LTL, while the individual effects of these health behaviors are modest (29). Such evidence highlights the importance of sufficiently intensive multidomain healthy lifestyle intervention for facilitating LTL maintenance.

Interestingly, there were more pronounced FINGER intervention benefits on LTL maintenance in APOE-ε4 carriers. The APOE-ε4 allele is the strongest known genetic risk factor for AD, and it has also been linked to other neurological and cardiovascular conditions (http://www.alzgene.org/). APOE-ε4 carriers also have a more rapid LTL attrition rate (30) and are more sensitive to the detrimental effects of unhealthy lifestyle (31). It has also been shown that among Alzheimer’s disease patients, those who were homozygous for APOE-ε4 had shorter LTL than those who were heterozygous for APOE-ε4 or noncarriers (32). Consistent with previous findings (33), the current FINGER LTL results showed that having the APOE-ε4 allele did not compromise intervention benefits in this genetically susceptible high-risk group. The results also showed that the FINGER intervention benefits on LTL maintenance were more pronounced among younger-old participants. LTL tends to shorten with advancing age (1), and it may be that lifestyle-related change in LTL is more effective among younger-old participants who have not yet undergone more extensive LTL shortening.

Several mechanisms have been suggested for LTL shortening, including, for example, oxidative stress, inflammation, chronic stress, and hyperactivation of the hypothalamic–pituitary–adrenal axis (34–36). These seem to be at least partly influenced by lifestyle-related factors (10). While the present study cannot pinpoint the exact mechanisms behind the observed effects on LTL change, the FINGER intervention targeted multiple lifestyle factors simultaneously, with a potential impact on several of these mechanisms.

Mechanisms related to LTL shortening have also been linked to pathophysiological processes in dementia-related conditions and cognitive decline (34), which may explain the associations between change in cognition and LTL change in this study. Our findings are in line with previous observational studies where shorter LTL was associated with Alzheimer’s disease and poorer cognitive performance in various domains (2,4,5,7,8). While FINGER participants with increasing LTL had significantly more intervention benefits on executive functioning and long-term memory, we cannot fully confirm whether such effects are domain-specific.

This study has some limitations. LTL was only measured in a subsample of FINGER participants, although this subsample was very similar to the rest of the target population. The FINGER trial was not powered to detect intervention effects on cognition by change in LTL. LTL was only measured at baseline and at the 2-year follow-up, limiting a more detailed assessment of change trajectories. For example, previous evidence suggested that while the annual LTL decline is linear among older adults, it is more accelerated after approximately age 69 years (37), which could not be assessed with our current measures. Although previous studies have shown inter/intra-individual LTL variation, as well as gradual longitudinal declines in LTL (37,38), it is difficult to directly compare the rate of LTL change in FINGER with previous reports. This is due to the different FINGER trial design, that is, older adults with a higher risk for dementia (according to the CAIDE risk score) selected for a multidomain lifestyle intervention to prevent cognitive decline. FINGER LTL measurements were conducted using previously published methods. However, there is no “general reference scale” for relative LTL values, which can vary between different studies, and may not be directly comparable between studies. This makes the size of the observed relative LTL changes more difficult to interpret. Also, the coefficient of variation value of 8.35% based on 5 genomic DNA control samples may be somewhat higher than in studies using larger numbers of genomic DNA controls.

Strengths of the study include the large sample of older individuals at risk for dementia, the multidomain lifestyle intervention with a long duration, carefully controlled LTL measurements pre- and post-intervention, and the comprehensive neuropsychological test battery. The 2-year FINGER trial did not have dementia as outcome, but indicators derived from the cognitive test battery were used. The ongoing extended follow-up will facilitate analyses of LTL changes and dementia incidence.

In conclusion, findings from the FINGER exploratory LTL substudy support the beneficial effects of a multidomain lifestyle intervention on LTL maintenance particularly among individuals genetically susceptible to Alzheimer’s disease, that is, APOE-ε4 allele carriers, younger-old individuals, and those who succeed in making healthier lifestyle changes. In addition, better LTL maintenance was associated with more pronounced intervention benefits on cognition in individuals at risk for dementia from the general population. LTL is a suitable biomarker for inclusion in lifestyle interventions and may aid in identifying subgroups that benefit from such interventions.

## Supplementary Material

glaa279_suppl_Supplementary_Tables
