# Supplementary material for: Telomere Length Change in a Multidomain Lifestyle Intervention to Prevent Cognitive Decline: A Randomized Clinical Trial
Source: J Gerontol A Biol Sci Med Sci. 2020 Nov 11;76(3):491–8. doi: 10.1093/gerona/glaa279 (PMC7907495; doi:10.1093/gerona/glaa279)
Supplement: glaa279_suppl_Supplementary_Tables [file glaa279_suppl_supplementary_tables.docx]

**Supplement** to Sindi, Solomon, Kåreholt et al, Change in telomere length in a 2-year multidomain lifestyle intervention to prevent cognitive decline in at-risk older adults (FINGER): a randomised controlled trial

**Table S1. Characteristics of the FINGER participants with and without available measurements for LTL change**

| **Characteristics at baseline** | **Total** | **LTL available** | **LTL not available** | **p** |
| --- | --- | --- | --- | --- |
|  | **n** | **n=756** | **n=504** |  |
| **Demographic characteristics** |  |  |  |  |
| Age at the baseline visit (years) | 1260 | 69.2 ± 4.7 | 69.4 ± 4.6 | 0.481 |
| Sex (women, %) | 1260 | 352 (46.6) | 236 (46.8) | 0.927 |
| Education (years) | 1258 | 10.1 ± (3.4) | 9.7 ± (3.5) | **0.039** |
| APOEε4 carriers (n, %) | 1175 | 244 (33.7) | 145 (32.2) | 0.583 |
| **Vascular factors** |  |  |  |  |
| Systolic blood pressure (mmHg) | 1249 | 139.0 ± 16.3 | 141.8 ± 15.9 | **0.003** |
| Diastolic blood pressure (mmHg) | 1249 | 80.2 ± 9.5 | 80.6 ± 9.4 | 0.482 |
| Serum total cholesterol (mmol/l) | 1255 | 5.2 ± 1.0 | 5.1 ± 1.0 | 0.472 |
| Serum HDL-cholesterol (mmol/l) | 1255 | 1.4 ± 0.4 | 1.4 ± 0.4 | 0.934 |
| Fasting plasma glucose (mmol/l) | 1257 | 6.1 ± 0.9 | 6.1 ± 0.9 | 0.705 |
| Body mass index (kg/m^2^) | 1249 | 28.1 ± 4.8 | 28.3 ± 4.6 | 0.484 |
| History of hypertension | 1246 | 481 (64.4) | 340 (68.1) | 0.172 |
| History of diabetes | 1253 | 101 (13.4) | 64 (12.8) | 0.099 |
| **Lifestyle factors** |  |  |  |  |
| Physical activity 2 or more times/week (%) | 1247 | 540 (72.0) | 343 (69.0) | 0.256 |
| Current smokers (%) | 1255 | 67 (8.9) | 44 (9.4) | 0.765 |
| Alcohol drinking at least once/week (%) | 1252 | 343 (45.7) | 213 (42.5) | 0.271 |
| Fish intake at least twice/week (%) | 1253 | 392 (52.1) | 264 (52.7) | 0.844 |
| Daily intake of vegetables (%) | 1257 | 480 (63.7) | 296 (58.9) | 0.085 |
| Recommended Finnish Diet Score points | 1253 | 12.8 ± 3.4 | 12.8 ± 3.4 | 0.985 |
| **Baseline Cognition*** |  |  |  |  |
| NTB total score | 1259 | 0.04 ± 0.6 | -0.07 ± 0.6 | **0.001** |
| Executive functioning | 1258 | 0.05 ± 0.7 | -0.11 ± 0.7 | **<0.001** |
| Processing speed | 1259 | 0.04 ± 0.8 | -0.07 ± 0.8 | **0.035** |
| Memory | 1259 | 0.03 ± 0.6 | -0.04 ± 0.7 | 0.067 |
| Complex memory | 1237 | 0.02 ± 0.7 | -0.03 ± 0.8 | 0.256 |

Values are means ± SD unless otherwise specified. Differences between groups with and without available LTL data were analysed with chi-square and t-tests as appropriate.

*Scores on the NTB total score, executive functioning, processing speed, memory and abbreviated memory are mean values of z scores of the cognitive tests included in each cognitive outcome. Higher scores indicate better performance.

**Supplementary Table S2. FINGER intervention effect on change in LTL, with and without adjustment for baseline LTL.**

|  | **Analyses adjusting for baseline LTL** | **Analyses not adjusting for baseline LTL*** |
| --- | --- | --- |
|  | Unstandardized β-coefficient (95% CI), p-value | |
| Intervention effects on LTL change | 0.007 (-0.015 - 0.030), p=0.53 | 0.002 (-0.024 - 0.028), p=0.87 |
| **Factors tested as effect modifiers: | | |
| APOEε4 allele | **0.054 (0.007-0.102), p=0.026** | **0.056 (0.002-0.110), p=0.042** |
| Baseline age | **-0.005 (-0.010 ‒ -0.001), p=0.031** | **-0.006 (-0.012 ‒ -0.0003), p=0.038** |
| Healthy lifestyle change index | **0.047 (0.005-0.089), p=0.029** | **0.056 (0.008-0.105), p=0.023** |
| Sex | -0.019 (-0.065-0.026), p=0.40 | 0.016 (-0.036-0.069), p=0.54 |

Unstandardized β-coefficients (95% CIs) and p-values are shown from linear regression models with change in LTL as dependent variable, and randomization group, age, sex, study site, baseline LTL, and healthy lifestyle change index as dependent variables. *Results shown from models not including baseline LTL.

** Unstandardized β-coefficients (95% CIs) and p-values are shown for the randomisation group x factor interactions (age and healthy lifestyle change index as continuous variables).

**Supplementary Table S3. The associations between change in LTL and change in cognition, with and without adjustment for baseline LTL.**

| Cognitive domain | **Analyses adjusting for baseline LTL** | **Analyses not adjusting for baseline LTL*** |
| --- | --- | --- |
|  | Estimate (95% CI), p-value | |
| NTB Total score | 0.127 (-0.011 – 0.264)  p = 0.070 | 0.127 (-0.011 – 0.264)  p = 0.070 |
| Memory | 0.152 (-0.074-0.378)  p=0.187 | 0.152 (-0.074-0.378)  p=0.187 |
| Executive function | **0.227 (0.057 – 0.396)**  **p = 0.009** | **0.227 (0.057 – 0.396)**  **p = 0.009** |
| Long-term memory | **0.257 (0.024 – 0.489)**  **p = 0.031** | **0.257 (0.024 – 0.489)**  **p = 0.031** |
| Processing speed | -0.087 (-0.268 – 0.094)  p = 0.347 | -0.087 (-0.268 – 0.094)  p = 0.347 |

Estimates (95% CIs) and p-values are shown for the randomisation group x time x LTL change interaction. Mixed effects regression models with maximum likelihood estimation were performed to assess the change in cognitive scores as a function of randomisation group, time, LTL change, their 2-way interactions, and a group x time x LTL change interaction. All models were adjusted for age, sex, study site, baseline LTL, and healthy lifestyle change index. *Results shown for models not including baseline LTL.
